# Spinal manipulation in the treatment of patients with MRI-confirmed lumbar disc herniation and sacroiliac joint hypomobility: a quasi-experimental study

**DOI:** 10.1186/s12998-018-0185-z

**Published:** 2018-05-17

**Authors:** Esmaeil Shokri, Fahimeh Kamali, Ehsan Sinaei, Farahnaz Ghafarinejad

**Affiliations:** 1 Physical Therapy Department, School of Rehabilitation Sciences, Shiraz University of Medical Sciences, Shiraz, Iran; 2 Rehabilitation Sciences Research Center, Shiraz University of Medical Sciences, Shiraz, Iran; 3 School of Rehabilitation Sciences, Shiraz University of Medical Sciences, Abiverdi 1 St,Chamran Blvd., P.O. Box 71345-1733, Shiraz, Iran

**Keywords:** Spinal manipulation, Lumbar disc herniation, Sacroiliac joint, Back pain

## Abstract

**Background:**

To investigate the effect of lumbar and sacroiliac joint (SIJ) manipulation on pain and functional disability in patients with lumbar disc herniation (LDH) concomitant with SIJ hypomobility.

**Methods:**

Twenty patients aged between 20 and 50 years with MRI-confirmed LDH who also had SIJ hypomobility participated in the trial in 2010. Patients who had sequestrated disc herniation were excluded. All patients received five sessions of spinal manipulative therapy (SMT) for the SIJ and lumbar spine during a 2-week period. Back and leg pain intensity and functional disability level were measured with a numerical rating scale (NRS) and the Oswestry Disability Index (ODI) at baseline, immediately after the 5th session, and 1 month after baseline.

**Results:**

A significantly greater mean improvement in back and leg pain was observed in the 5th sessions and 1 month after SMT. Mean changes in ODI in the 5th session and 1 month after treatment also showed significant improvement. The MCIC for NRS and ODI scores in the present study were considered 20 and 6 points, respectively. Therefore, the mentioned improvements were not clinically significant in the 5th session or at 1-month follow-up.

**Conclusion:**

Five sessions of lumbar and SIJ manipulation can potentially improve pain and functional disability in patients with MRI-confirmed LDH and concomitant SIJ hypomobility.

**Trial registration:**

Irct.ir (Identifier: IRCT2017011924149N33), registered 19 February 2017 (retrospectively registered).

## Background

Common low back problems include disc prolapse, spinal stenosis and low back pain [1]. Disc herniation can be categorized as protrusion (disc contained by the annulus fibrosus), extrusion (disc materials migrated out through the annulus fibrosus, but contained by the posterior longitudinal ligament) and sequestration (disc materials released into the spinal canal) [1]. Disc prolapse commonly presents with pain and numbness radiating to the buttocks and legs due to spinal nerve or nerve root compromise; however, it may be asymptomatic in approximately 24% of all cases [1]. Symptomatic lumbar disc disease (SLDD) is a term used to differentiate between structural abnormalities without clinical symptoms and abnormalities that induce clinical presentations [2]. Approximately 95% of all instances of lumbar disc herniation (LDH) occur at L4-L5 and L5-S1 levels [1].

Lumbar disc herniation commonly presents with low back pain, and this problem is usually associated with sacroiliac joint (SIJ) disorders. In fact, up to 30.7% of patients with LBP and sciatica also have SIJ dysfunction [3]. A recent study reported the prevalence of SIJ dysfunction as 72.3% among patients with LDH [4]. Researchers have claimed that depending on the type of SIJ disorder, the lumbar spine (mostly L5) can also be involved [5]. The SIJ is part of the lumbar– pelvic–hip complex; since this complex works as a mechanical unit, the involvement of any structure can affect the position and movement of other sections [6].

Generally speaking, most patients with SLDD prefer conservative treatments to surgical intervention. To date, evidence has supported several conservative treatments for SLDD including traction [7], McKenzie extension exercises [8] and rehabilitation [9].

The use of spinal manipulative therapy (SMT) for patients with SLDD has also been suggested; however, its safety and indications have remained debatable, particularly in individuals with disc disruption or instability [2]. In this regard, the risk of SMT causing clinically worsened disc herniation or cauda equine syndrome in patients with LDH is estimated to be less than one in 3.7 million [2]. A systematic review in 2004 also confirmed the safety and effectiveness of SMT for patients with SLDD [2].

Some studies reported significant clinical improvements in patients with SLDD after manipulation in comparison to traction [10], heat [11] and sham manipulation [12], but no significant differences when compared to exercise therapy and medical corsets [11]. A recent study found long-term improvement in pain and functional activity after 1 year of follow-up [13], and another study in 2016 reported significant improvement in leg pain after 1 month in patients with extrusion and sequestration of lumbar discs, following manipulation [14].

Sacroiliac joint hypomobility has usually been overlooked in the management of patients with LDH and low back problems. However, there is no conclusive evidence for the effectiveness of SMT in the treatment of patients with LDH, and the evidence to date is contradictory. Therefore, the present study aimed to investigate the effect of SMT applied to the lumbar spine and SIJ to treat patients with SLDD who also had SIJ hypomobility.

## Methods

### Participants

Twenty patients (11 males, 9 females) aged 20–50 years old with MRI-proven unilateral LDH were included IN 2010 if they had SLDD in the L4-L5 or L5-S1 segment concomitant with ipsilateral SIJ hypomobility (Table 1). Leg pain during 1 to 10 months before the study was their major complaint, and the mean level of leg pain during the previous 24 h was 30–70 out of 100 on a 0–100 numerical rating scale (NRS). The time interval allowed between the MRI diagnosis and inclusion in the study was 3 months. Exclusion criteria were sequestrated LDH with neurological signs, spinal canal stenosis, spondylolisthesis, previous lumbar surgery and gross instability. Patients a with positive *well straight leg raise (SLR) test,* indicating rather large disc herniation and poor prognosis for conservative treatments [15, 16], were also excluded.

**Table 1:** Participants’ demographic characteristics (*N* = 20)

Variable		Value^a^
Age		37.86 ± 9.62
BMI		25.10 ± 3.12
Side of LDH	Right	11 (55)
	Left	9 (45)
Side of SIJ hypomobility	Right	8 (40)
	Left	12 (60)
Segment of LDH	L4 - L5	5 (25)
	L5 - S1	15 (75)

*Abbreviations*: *BMI* body mass index, *LDH* lumbar disc herniation, *SIJ* sacroiliac joint

^a^Values are mean ± SD for continuous variables and number (percentage) for categorical variables

### Study design

This was a pre–post test quasi-experimental study. The participants were selected among patients referred to physical therapy clinics of Shiraz, Iran. Sample size was calculated based on the NRS pain score reported in a previous related study (mean [95% CI] = 22 [15–30], α = 0.05, β = 0.02) [17]. Written informed consent was obtained and ethical approval was granted by the *Shiraz University of Medical Sciences* Ethics Committee (approval number CT-88-4614).

Demographic data, pain intensity, functional disability and clinical diagnostic tests were recorded at baseline. After that, the patients received five sessions of manipulative therapy on alternate days, and the outcomes were reassessed after the 1st and 5th sessions and at a 1-month follow-up. All patients received both lumbar and SIJ manipulations in each treatment session.

### Interventions

#### Lumbar rotation manipulation

**Fig. 1:**
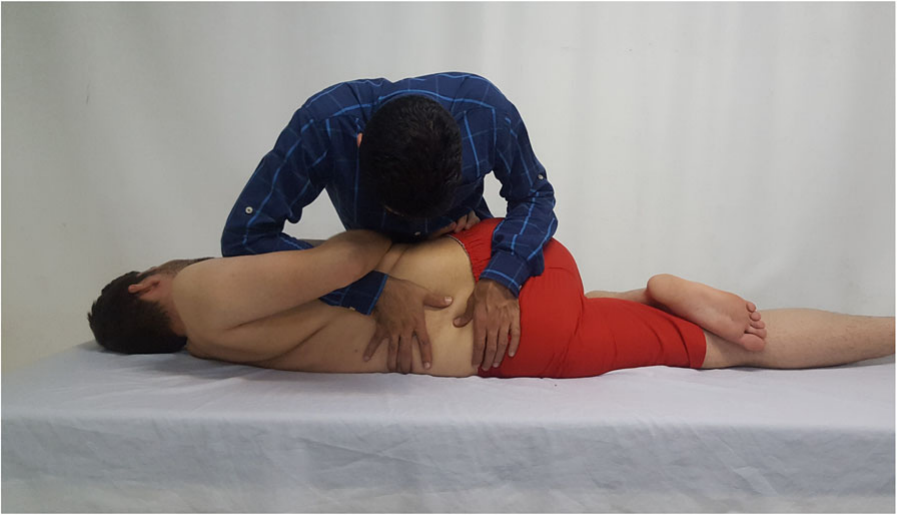
Lumbar rotation manipulation

The neutral position of the spine was used for side-posture lumbar manipulation. The patient lay on the asymptomatic side (e.g. left) in the lateral recumbent position, with his or her upper foot in the popliteal fossa of the lower leg. Standing opposite to the patient, the therapist grasped the patient’s lower shoulder and arm and applied right rotation until motion was felt in the desired segment of the lumbar spine. The patient was rolled toward the therapist, with his or her arms positioned around the therapist’s right arm. The therapist’s right forearm was in contact with the patient’s right axilla and pectoral region to maintain appropriate rotation from above. In this position, the therapist applied a high-velocity low-amplitude thrust to the pelvis in an anterior direction with his or her left forearm placed behind the patient’s right hip. The therapist pressed the spinous process of the upper vertebra downward with his or her right thumb, while pulling the spinous process of the lower vertebra upward with his or her left thumb. The procedure was done in a way that avoided exacerbating the patient’s pain at the barrier point [18] (Fig. 1).

#### Sacroiliac joint manipulation

**Fig. 2:**
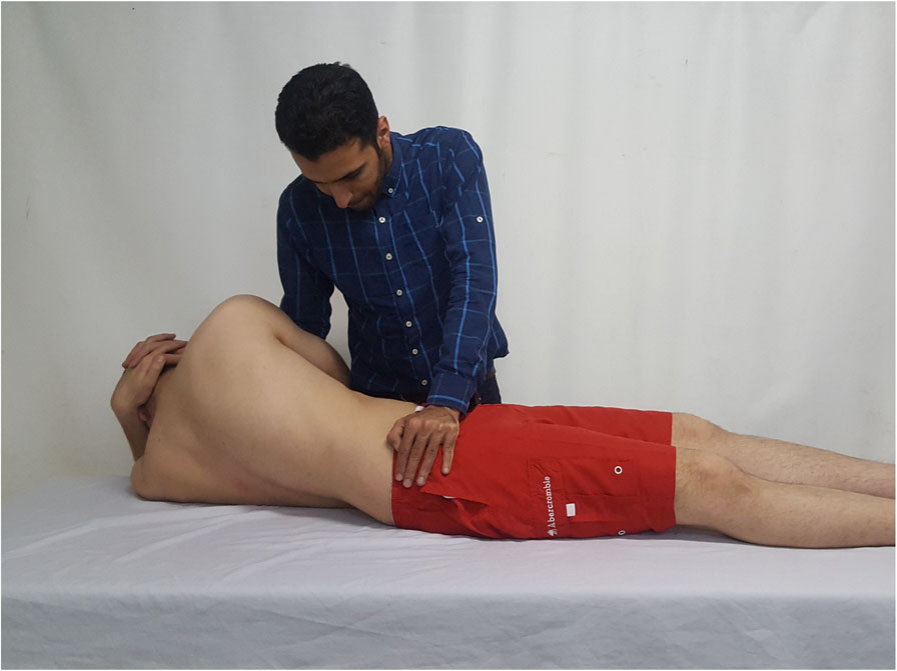
Sacroiliac joint manipulation

The patients lay supine on a treatment table, with their fingers interlocked behind their head. The therapist stood contralateral to the side to be manipulated and moved the patient onto his or her side, then leaned toward the dysfunction side, rotated the patient, and exerted a quick thrust to the anterior superior iliac spine in the posterior and inferior directions [18, 19] (Fig. 2).

### Outcome measures

The scores for back and leg pain were recorded separately on an NRS of 0 to 100, in which 0 indicated no pain and 100 indicated the worst perceived pain [20]. The minimal clinically important change (MCIC) in NRS score was 20 points [21, 22].

The patients’ functional disability level was determined with the Oswestry Disability Index (ODI), a 10-item questionnaire in which each item is scored from 0 to 5 [23]. The maximum score on the ODI is 50, and higher scores indicate greater functional disability. However, in the present study the maximum score was 45, since the sex life item was omitted due to cultural issues. Therefore relative values are reported here as the total score/total possible score × 10. The MCIC for the ODI was reported as 6 points in a sample of patients with LBP who received physical therapy [24].

Participants were also assessed with the SLR and slump tests to diagnose LDH, and standing flexion, sitting flexion and Gillet tests were used to diagnose SIJ hypomobility. Although the evidence is contradictory, some studies have reported acceptable reliability for the SIJ test. [25–27] The results of five clinical tests were recorded as positive or negative values at baseline, in the 5th session and 1 month after baseline.

### Statistical analysis

The data were analyzed with the Statistical Package for Social Sciences (SPSS), version 21.0 (IBM Corp., Armonk, NY, USA). The Kolmogorov–Smirnov test of normality was conducted for all quantitative variables. Repeated measure ANOVA was used to assess the trends in changes in the NRS and ODI scores. Individual time point differences were determined by the Bonferroni post hoc test, and the results of the five clinical tests were analyzed with the McNemar test.

## Results

**Fig. 3:**
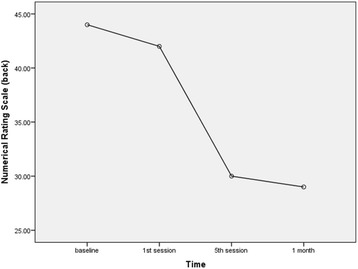
Trend in back pain intensity during the trial

**Fig. 4:**
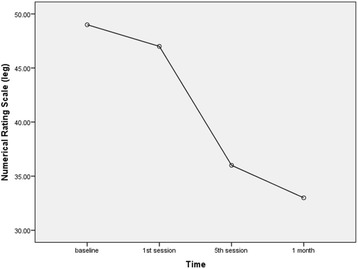
Trend in leg pain intensity during the trial

The NRS score for back pain showed statistically significant improvement in the 5th session (*P* = 0.034) and at 1-month follow-up (*P* = 0.047) compared to the baseline value. In addition, statistically significant improvement in the leg NRS score was seen in the 5th session (*P* = 0.010) and at 1-month follow-up (*P* = 0.006). Because the MCIC for NRS scores in the present study was 20 points, NRS score improvements in back and leg pain were not clinically significant in the 5th session or at 1-month follow-up (Figs. 3 and 4) (Table 2).

**Table 2:** Mean values of back pain, leg pain and functional disability during the trial

Outcomes	Baseline^a^	1st session	5th session	1 month	Times with statistically significant differences (*P*-value)
Back NRS	44.95 ± 26.18	42.00 ± 25.30	30.50 ± 19.32	29.75 ± 16.42	Baseline - 5th session (0.034)
					Baseline - 1 month (0.047)
Leg NRS	49.50 ± 23.94	47.25 ± 24.35	36.00 ± 16.90	33.75 ± 13.75	Baseline - 5th session (0.010)
					Baseline - 1 month (0.006)
ODI	14.45 ± 4.40	n.d.	11.35 ± 4.54	10.95 ± 4.27	Baseline - 5th session (0.001)
					Baseline - 1 month (0.001)

*Abbreviations*: *NRS* numerical rating scale, *ODI* Oswestry Disability Index, *n.d.* not determined

^a^Values are mean ± SD

**Fig. 5:**
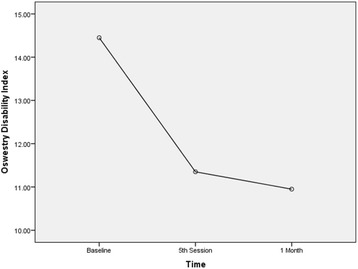
Trend in functional disability level during the trial

The ODI scores indicated statistically significant improvement in the 5th session (*P* = 0.001) and at 1-month follow-up (P = 0.001). Because the MCIC for ODI score in the present study was 6 points, the improvements were not considered clinically significant at the 5th session or at 1-month follow-up (Fig. 5) (Table 2).

In 78.3% of our patients, the sides of SIJ hypomobility and LDH were the same. After treatment, the patients showed statistically significant improvements in Gillet, standing flexion and sitting flexion tests in the 5th session and at 1-month follow-up (*P* ≤ 0.001). The findings also indicated 95% improvement in the results of the SIJ hypomobility tests after SMT. We also observed a 20% improvement in the SLR test results and a 15% improvement in the slump test in the 5th session and at 1-month follow-up after SMT, although these changes were not statistically significant (Table 3).

**Table 3:** Distribution of improvements in diagnostic test results in the 5th session and after 1-month follow-up

Test	5th session		1 month	
	(Positive/Negative)	*P*-value	(Positive/Negative)	*P*-value
Gillet	1/19	< 0.001^*^	1/19	< 0.001^*^
Standing flexion	1/19	< 0.001^*^	1/19	< 0.001^*^
Sitting flexion	1/19	< 0.001^*^	1/19	< 0.001^*^
SLR	16/4	0.12	17/3	0.25
Slump	17/3	0.25	17/3	0.25

*Abbreviations*: *SLR* straight leg raising

^*^Significant recovery compared to baseline

## Discussion

The aim of the present study was to investigate the effect of SMT on pain, functional disability and the results of clinical tests of SIJ function and LDH in patients with unilateral SLDD plus SIJ hypomobility. Our findings suggest that five sessions of lumbar and SIJ manipulation can lead to statistically significant improvement in pain and functional disability, which in turn may restore normal SIJ mobility in these patients.

Compared to common treatments for LDH, SMT is reported to be 37,000 to 148,000 times safer than nonsteroid antiinflammatory drugs and 55,500 to 444,000 times safer than surgery [28]. Neither worsening of symptoms nor cauda equine syndrome were observed in our participants after SMT. Epidemiologic data on the rate of injuries caused by manipulation are limited. The most common incidents are related to innocuous physiologic reactions or short-term discomfort generated at the treatment site. However, these are self-limiting events that usually resolve within 24 h after SMT [28].

In rotational side-posture lumbar manipulation, the impact of the facet joints limits axial rotation of the lower lumbar vertebrae and consequently prevents annulus fibrosus tearing [28]. In the present study, patients with sequestrated LDH who had neurological signs were excluded because these patients may have bowel and bladder disorders, and many (but not all) of them are thus candidates for surgery [29]. In the present study manipulation was applied in the neutral flexion–extension position to reduce the risk of injury.

The diagnosis and treatment of SIJ hypomobility in patients with SLDD are important issues that have not been adequately addressed in the literature. In 78.3% of our cases, the side of SIJ hypomobility was the same as the side of LDH. After treatment, 95% improvement was obtained in the results of SIJ hypomobility tests (Table 2).

The SIJ has been reported to be one of the main sources of low back disorders [5]. A recent study also found that SIJ dysfunction was a prevalent concomitant pathology in patients with LDH. Therefore, SIJ dysfunction should be considered in the treatment of these patients [4]. Pelvic asymmetry as well as hypermobility or hypomobility of the spinal or sacroiliac joints can cause low back pain [3, 30]. Any involvement of the SIJ can induce muscle spasm in the piriformis, which in turn can lead to sciatic irritation and a wide range of symptoms mimicking radiculopathy [5]. Increased tension in the quadratus lumborum, iliopsoas or hamstring muscles may also affect the SIJ mechanism of action. Presumably, SIJ manipulation can decrease tension in these muscles and consequently correct lumbar spine dysfunction [3, 31].

Several mechanisms have been theorized for the mechanical and neurophysiological basis of SMT, including stimulation or modulation of the somatosensory system to evoke neuromuscular reflexes [32]. Forceful stretching of the spinal muscles induces relaxation after SMT. Other mechanisms are induced hypoalgesia [33], kinematic correction [34, 35] and increased lumbar range of motion [36]. A brief reduction in intradiscal pressure during SMT in cadavers and return to baseline within less than 1 min was reported in one earlier study [37]. Another study showed reduced H-reflex amplitude in patients with unilateral disc herniation, which improved after SMT [11]. The improved outcomes in our patients can be attributed to two main factors. Firstly, SIJ manipulation may improve normal functioning of the lumbar spine and related muscles [38]. Secondly, lumbar side-posture rotational manipulation can induce spinal muscle relaxation [32], improve lumbar range of motion [36], and briefly decrease intradiscal pressure [37].

The results of the SIJ hypomobility tests (including the Gillet test, standing flexion and sitting flexion tests) improved significantly in the 5th session and after 1-month follow-up compared to baseline values, whereas no statistically significant improvement was observed in the SLR and slump tests. Spinal manipulative therapy may enhance mobility of the SIJ and lumbar vertebrae, and affect the muscles in these regions, thus accounting for the improvement in pain and functional ability. However, significant changes in the slump and SLR tests may require additional interventions such as soft tissue manipulation and nerve mobilization, which were not tested in this study.

In one controlled trial, SMT and sham manipulation were compared in 102 participants with MRI-confirmed LDH; the SMT group showed significantly greater improvement in back and leg pain after 6 months [12]. Nevertheless, the intervention in that study was a combination of soft tissue manipulation and thrust manipulation, and the diagnosis and treatment of SIJ hypomobility were not considered.

In a prospective cohort study, Leemann et al., investigated the effect of high-velocity, low-amplitude SMT in patients with acute or chronic MRI-confirmed LDH, and reported clinically significant improvement in back and leg NRS and ODI scores in both short-term and long-term assessments [13]. In a follow-up study, Ehrler et al., investigated the association of magnetic resonance imaging features, including axial location and type of herniation, with the outcomes of SMT in patients with LDH [14]. This study reported greater improvements in symptoms among patients with sequestrated SLDD who received SMT to the level of herniation. These studies, however, did not consider the treatment of the SIJ in patients with LDH.

Burton et al., also compared SMT with chemonucleolysis in the treatment of patients with SLDD, and reported greater improvements in back pain and disability in the first few weeks in the SMT group [39]. Their SMT, however, included a combination of thrust manipulation, mobilization and soft tissue stretching.

The results of previous studies have shown that SMT is effective in the treatment of LDH [40]. The study most similar to ours is the one by Galm et al., which included 150 patients with LDH, 46 of whom had SIJ dysfunction. All participants received routine physiotherapy, mobilization and SMT in the prone position. Significant improvements were reported in lumbar and ischiatic pain in the SIJ dysfunction group. These authors concluded that in the presence of lumbar and ischiatic symptoms, appropriate treatment for SIJ dysfunction should be considered regardless of intervertebral disc pathomorphology [3]. In this study, however, the number of treatment sessions and the results of SIJ and LDH physical tests were not reported.

Our study had some limitations which should be noted. The pre–post test design did not include a control group; consequently, the results cannot be considered evidence in support of the clinical efficacy of SMT for patients with LDH and SIJ hypomobility. A controlled trial is advisable in which combined manipulations are compared to lumbar or SIJ manipulation separately, to elucidate whether using both lumbar and SIJ manipulation together yields better outcomes than using a single type of manipulation. The small sample size and lack of long-term follow-up are other limitations. In addition, we are aware that measuring physiologic responses to SMT by recording electromyographic activity of the spinal muscles, the myotomes and dermatomes of the involved nerve roots, would strengthen the results of future studies. Also, more reliable tests for SIJ dysfunction are available and should be used in future studies. Despite these limitations, we addressed some shortcomings of previous studies. The strengths of the present study were matching of the physical examination findings with imaging findings, considering SIJ hypomobility in the treatment of patients with LDH, and the application of spinal thrust manipulation alone rather than a combination of therapeutic methods.

## Conclusions

Spinal manipulative therapy can potentially improve pain, functional disability and SIJ mobility in patients with LDH concomitant with SIJ hypomobility; therefore, it can be implemented in physical therapy programs for these patients. However, further studies with larger sample sizes, longer follow-up periods and real control groups should be done to provide more accurate results.

## Abbreviations

LDH: Lumbar disc herniation
MCIC: Minimally clinically important change
NRS: Numerical rating scale
ODI: Oswestry disability index
SIJ: Sacroiliac joint
SLDD: Symptomatic lumbar disc disease
SMT: Spinal manipulative therapy
